# Supplementary material for: The compounding effect of having HIV and a disability on child mortality among mothers in South Africa
Source: PLoS One. 2021 May 5;16(5):e0251183. doi: 10.1371/journal.pone.0251183 (PMC8099123; doi:10.1371/journal.pone.0251183)
Supplement: Attachment — Submitted filename: Response to Reviewers.docx [file pone.0251183.s001.docx]

**RESPONSE TO REVIEWERS**

**EDITOR COMMENTS:**

**COMMENT #1**: The fact that you there are no authors from South Africa who have a good understanding of the issues at hand in that country, I find it very difficult to believe that your research is meaningful and policy relevant in the country from where the data was collected.

**RESPONSE #1:**

Thank you for your comment. We really appreciate and fully support the idea of “Nothing about us without us.” We have invited Dr. Mark Blecher, Chief Director of the Health and Social Development at the National Treasury of South Africa to review our paper and contribute by highlighting the policy relevant issues from South Africa government perspective. Dr. Mark Blecher contribution was significant and we have added him as a co-author on this paper.

**COMMENT #2:** What are the policy relevant issues from your study from a south Africa government perspective.

**RESPONSE #2:**

Thank you for your comment. With the help from Dr. Mark Blecher we have addressed this in the text of the manuscript. We have now added a separate sub section “Policy implication” to the Discussion section and we suggest that the “South African government, donors, and NGOs may use findings from this study to measure progress in ongoing policy and program implementation efforts” in addressing the needs of women with disabilities, especially disabled women with HIV infection.

**COMMENT #3:** What are the policy implications for the SADC region as a whole? The research is just an academic exercise and has no meaning if these fundamental questions are not answered by the researchers.

**RESPONSE #3:**

Thank you again for your comment. We have now addressed this in the Policy implications section. For example, we have stated that, “This study’s findings provide empirical evidence that South African women with disabilities are sexually active and have children at rates comparable to those of their non-disabled peers. The inclusion of disability in the national strategic plans and a more disability-inclusive HIV service ecosystem in South Africa does not yet equally benefit women with disabilities, especially women with disabilities with HIV infection.”

**REVIEWER # 1 (DR. GRANT MUREWANHEMA):**

**COMMENT #1:** A scientifically sound and well-thought piece of academic looking at the effects of having HIV and a disability on child mortality among mothers in South Africa. The statistical methods, data interpretation and discussion are well-laid out in a clear way.

**RESPONSE #1**: Thank you for taking your time to review our manuscript.
